# Laparoscopic Surgical Techniques for Endometriosis and Adenomyosis

**DOI:** 10.1155/DTE.6.153

**Published:** 2000

**Authors:** C. Wood, P. Maher, R. Woods

**Affiliations:** 1 Endometriosis Care Centre of Australia Monash IVF Monash University Cliveden Hill Private Hospital Victoria Australia; 2 Mercy Hospital for Women Victoria Australia; 3 Box Hill Hospital Melbourne Victoria Australia; 4 19 Simpson Street Melbourne Victoria 3002 Australia

## Abstract

The details of surgical techniques for laparoscopic removal of endometriosis and adenomyosis are described briefly in textbooks and gynaecological journal articles. We have described a wide variety of techniques for the various procedures required in the treatment of endometriosis and adenomyosis, excluding hysterectomy. The principles are based upon those used in removal of primary cancer lesions. The limitations of thermal ablation are discussed, and evidence of improved results after excision of lesions have been submitted for publication.


*Diagnostic and Therapeutic Endoscopy*, Vol. 6, pp. 153–168
Reprints available directly from the publisher
Photocopying permitted by license only

© 2000 OPA (Overseas Publishers Association) N.V.
Published by license under
the Harwood Academic Publishers imprint,
part of The Gordon and Breach Publishing Group.
Printed in Malaysia.

### Laparoscopic Surgical Techniques for Endometriosis and Adenomyosis

C. WOOD[a,*], P. MAHER[b] and R. WOODS[c]

[a]*Endometriosis Care Centre of Australia, Monash IVF, Monash University, Cliveden Hill Private Hospital, Victoria, Australia;* [b]*Mercy Hospital for Women, Victoria, Australia;* [c]*Box Hill Hospital, Melbourne, Victoria, Australia*

*(Received 25 January 2000; Revised 10 March 2000; In final form 10 May 2000)*

**The details of surgical techniques for laparoscopic removal of endometriosis and adenomyosis are described briefly in textbooks and gynaecological journal articles. We have described a wide variety of techniques for the various procedures required in the treatment of endometriosis and adenomyosis, excluding hysterectomy. The principles are based upon those used in removal of primary cancer lesions. The limitations of thermal ablation are discussed, and evidence of improved results after excision of lesions have been submitted for publication.**

*Keywords:* Adenomyoma, Endometriosis, Laparoscopy, Surgical techniques

### INTRODUCTION

A review of surgical treatment of endometriosis is presented because of the difficulty expressed by gynaecologists in removing endometriosis when it is extensive or involving vital structures such as the ureter, bladder, rectum, ovary or major vessels. This has been exemplified by a study of 198 patients with recurrent endometriosis referred to the Endometriosis Clinic with an average of 5.7 previous medical and surgical treatments who underwent further laparoscopy assessment [1]. Two-thirds of these patients had disease mainly located over the course of the ureter, or on the rectum, the vagina, or the bladder. This suggests that the previous surgery, which involved $CO_2$ laser or diathermy and was performed on an average 2.7 times, may have avoided vital structures to avoid the risk of trauma to them.

Our previous articles concerning the use of laparoscopy surgery have not detailed the surgical techniques. The difficulty in learning laparoscopic techniques is illustrated by a review of journal articles and books describing techniques used for the laparoscopic removal of an ovary. The number of steps described was always 6 or less and the detailed pieces of information which make this a safe procedure were always less than 20. In our own estimation there were 19 important steps and over 100 pieces of relevant information (see text).

* Corresponding author. 19 Simpson Street, Melbourne, Victoria 3002, Australia. Tel.: +61 3 9415 7722. Fax: +61 3 9415 8461. E-mail: profwood@malvern.hotkey.net.au.

Endometriosis is the most frequent reason for gynaecologic operative laparoscopy in the United States and probably also in Australia [2]. It is therefore important for the laparoscopist to be thoroughly familiar with the current standards of management.

TeLinde and Scott defined the objectives of surgical treatment of endometriosis in 1952: "one should excise or fulgurate all evident endometriosis." The surgical objectives of laparoscopic treatment are similar, i.e. to remove all evident endometriosis by excising large superficial and deep lesions and only vaporizing smaller deposits. The limited hormonal responsiveness of ectopic endometrium has determined the need to perform surgery [3].

The technical advantages of a laparoscopic approach to endometriosis surgery include: easy intraoperative access to the rectum, vagina, and ureter, magnification which is easier to manipulate than an operative microscope, and the ability to perform an underwater examination at the end of the procedure during which all blood clot is evacuated and complete haemostasis obtained. The general advantages of laparoscopy include: same day diagnosis and treatment, short hospitalization, rapid recuperation, superior scar cosmetics, excellent patient acceptance, cost effectiveness, and results at least equal to laparotomy [2].

The advanced laparoscopic surgeon may use a variety of techniques and requires equipment for mechanical cutting, electrosurgery, aquadissection, suturing and stapling. Electrosurgery and laser have equivalent results in the relief of pain and infertility [4].

### LAPAROSCOPIC TECHNIQUES

### Bleeding

Bleeding problems are common and difficult to resolve. Coagulation current is rarely used. The tip of a spoon electrode is used to cut and, the round body of the electrode is used to tamponade arteriolar bleeding vessels after which cutting current is applied to coagulate the vessel wall. The Kleppinger bipolar forceps (Richard Wolf) are most effective for large vessel haemostasis. A number of bipolar forceps are not robust and do not tamponade larger vessels so that the application of current is ineffective.

### Suturing

Suturing with large curved needles using 5 mm lower quadrant incisions requires a special technique to put them into the peritoneal cavity [5]. Lower abdominal incisions placed lateral to the rectus muscle make a path that is very easy to re-enter on removing the trocar sleeve. To suture with a needle, the trocar sleeve is taken out of the abdomen and loaded by introducing a grasping forceps through the cannula. The forceps grasps the distal end of the suture, and pulls the suture through the trocar sleeve. The forceps is reinserted through the cannula, and grasps the suture 2 cm from the needle. The needle and forceps is inserted into the peritoneal cavity through the original tract as visualized on the monitor; the needle follows through the soft tissue and the trocar sleeve is pushed downward over the driver to reinsert it at its original position in the peritoneal cavity.

The needle is grasped by the forceps and suturing achieved. Arter this the needle is placed in the anterior abdominal wall parietal peritoneum for removal after the suture is tied. The suture is cut adjacent to the needle, and the cut end of the suture is pulled out of the peritoneal cavity; the knot then is tied with a knotpusher without loss of pneumoperitoneum because of the tight seal of the trocar sleeve. The surgeon holding both strands makes a simple half-hitch. The knot-pusher is placed on one strand of the suture just above the knot, the suture is held firmly across the index finger and the throw is pushed down to the tissue defect. The second throw is made in the same direction (i.e. a slip knot) while exerting tension from above to further secure the tissue. The knot is secured with the third and fourth throws by pushing half-hitches, made in the opposite way, down to the knot to secure it firmly. To retrieve the needle the trocar sleeve is pulled out and the needle holder inside it drags the needle through the soff tissue. The trocar sleeve is replaced easily with or without another suture.

Suturing may be easier using the Endo Stitch®. It enables a suture to be placed with a 10 mm diameter instrument containing the needle and suture within the caliber of the instrument. Placement of the suture and the resultant tie are controlled from the handle avoiding the complexity of several separate steps using separate needle holding, suture needle and knot pushing equipment.

### Equipment

High flow $CO_2$ insufflation to 15 L/min or more is necessary to compensate for the rapid loss of $CO_2$ during suctioning. The ability to maintain a relatively constant intra-abdominal pressure between 10 and 15 mmHg during laparoscopic surgery is essential. Operating room tables capable of a 30° Trendelenburg's position are extremely valuable for laparoscopic deep pelvic dissection.

Extracorporeal tying is facilitated by using a trocar sleeve without a trap to avoid difficulty in slipping knots down to the tissue. The Apple Trocar® (Apple Medical, Boston, MA) has a tight seal preventing loss of pneumoperitoneum when pushing the knot down.

### Preoperative Preparation

Preoperative ovarian suppression is not often used for therapy as only mild endometriosis reduction occurs and that which does prevents the removal of residual microscopic lesions [3].

Preoperative oral Fleet® is given in 1–2 bottles if deep pelvic endometriosis is expected which rapidly clears the bowel. Alternatively, a Durolax® or Fleet® enema may be given to clear the lower bowel.

### PERITONEAL EXCISION

Our preference for excision is based upon the fallacy of vaporizing or burning lesions and then claiming endometriosis has been treated. The diagnosis of endometriosis is histological, not visual. Endometriosis is found in only 47% of yellow brown defects, 67% of glandular blister lesions and 81% of white opaque and red flame lesions [6,7]. Even in the most experienced hands visual identification is only 81% accurate, and in less experienced hands, 41% [8]. Biopsy studies show that at least 7% of lesions are missed and the extent of disease is underestimated in 50%, histology showing disease at the border of the excised tissue which was intended to be complete [9]. It is unfair and unethical to label a patient as having endometriosis when they may not, as unnecessary anxiety, possible drug therapy, and misdiagnosis is to their disadvantage. If this argument is accepted there is no point in taking a biopsy of part of the lesion, and burning the remainder, as biopsy necessitates complete excision. When multiple lesions with macroscopic features of endometriosis are present, only 2 of 3 lesions excised from 30 patients were histologically proven to be endometriosis. The remainder were granulomas, scar or vascular tissue [10]. The presence and extent of the disease can only be assessed by excising all suspicious lesions.

A controlled trial to compare peritoneal excision to thermal ablation is difficult to design as the diagnosis of endometriosis when all suspicious lesions are excised will be more accurate than vaporization or thermal ablation, when most of the tissue is not examined, and women without endometriosis will be included in the latter group.

Surgical considerations also favour excision. While most endometriosis is not penetrating, a significant number of women with persistent disease have lesions infiltrating beyond 5 mm, 21% (41 of 198) and 8% (16 of 198) infiltrating the rectal muscle and 8% (16 of 198) infiltrating the wall of the bladder [1]. The reason for the persistence or recurrence is most likely the use of thermal ablation when the depth of ablation may be limited because of either the false assumption that lesions are superficial and deeper treatment is unnecessary, or that harm may result to the bladder, bowel, ureter, arteries or veins.

The average number of previous surgical treatments by laser or electrocoagulation, was 2.7, so it is reasonable to assume that some of the 16 patients requiring resection of the rectum or excision of a segment of the bladder may have avoided this by earlier excision defining and removing the infiltrating lesion. Ten of these patients had symptoms recurring within 6 months of the previous laparoscopic treatment.

Occult microscopic endometriosis has been found in 13% of normal looking peritoneum next to the visible lesion [11]. This finding and the histologically proven frequent incomplete removal in 50% of cases when the edge of excised lesions were examined suggests that peritoneal excision or thermal ablation need to include 1 or 2 cm of tissue beyond the visible lesion [9].

Another limitation of thermal ablation is that if the pathology is coagulated it may be replaced by white scar which obscures assessment of the extent of pathology. Vaporization by laser or sparking point diathermy avoids this. This is achieved by using 80 W cutting current with a fine needle held 1–3 mm from the surface of the lesion. The use of thermal ablation may increase the risk of damaging the ureter, uterine artery, bladder or rectal muscle when the lesions are sited over the surface of these structures. The most common sites of endometriosis in the 198 patients with recurrent endometriosis was on the lateral pelvic wall over the course of the ureter and on the surface of the bladder, rectum, vagina and pararectum, accounting for 67% of cases. Excision allows safe removal of endometriosis over or attached to the ureter and precise excision of infiltrating lesions in the wall of the bladder, vagina and rectum.

Other reasons for using excision is the cost reduction as laser is not required, and the absence of necrotic areas which may form scar tissue which results from extensive electrocoagulation.

Although studies of excisional surgery are uncontrolled the lower recurrence rates reported after 1–3 years than after ablation suggests that it is equal or better [12–14]. The unsatisfactory diagnostic basis of a controlled trial of the 2 methods (see p. 7) limits proof that excision may be better than thermal ablation.

### LATERAL PELVIC WALL

Lateral pelvic wall peritoneum is mobilized before excision. Fine toothed grasping forceps are used to grasp the endometriotic lesion and retract the peritoneum medially from the lateral pelvic wall. If the lesion is friable, opaque or adherent to underlying tissue, normal peritoneum above the lesion is retracted and the dissection commenced in normal tissue enabling the size and depth of lesion to be defined. Surrounding anatomy such as the ureter, internal iliac artery and its branches becomes more obvious after peritoneal mobilization. The laparoscope is placed close to the lesion to magnify the anatomy. Blunt ended scissors or spoon electrode are used to open a small hole in the peritoneum. It is important to reflect any tissue adherent to the inner surface of the peritoneum as it may contain an adherent ureter or uterine artery, before extending the peritoneal excision. If the peritoneum is vascular, cutting or coagulation diathermy is used as required.

An elliptical incision is made in normal peritoneum surrounding the fibrotic portion of the lesion, its edge lifted outwards, and the lesion undermined using scissors or pressurized irrigant from a suction-irrigation device (aquadissector) to push the fibrotic endometriosis from the underlying pelvic side wall, rectum, or bladder. Gentle aquadissection opens low resistance tissue planes avoiding trauma to vessels on the ureter and is particularly useful if scar tissue prevents identification of normal anatomy. Suction should not be applied when the instrument is in contact with the ureter or blood vessels. This makes undercutting of the lesion with scissors or electrosurgery safer.

In the presence of peritoneal thickening it may be difficult to visualize the course of the ureter; the peritoneum is cut from the upper pelvic wall in 2–5 mm steps and the course of the ureter is identified as the peritoneum is retracted. Small blood vessels close to the ureter are avoided where possible as they may affect ureteric integrity. If the peritoneum is adherent to the ureter then the ureter is isolated proximal to the adherence where it is mobile, and retracted. This allows more control and accurate dissection between the ureter and peritoneum over the adherent area as the dissection proceeds forward.

If the ureter cannot be seen even after peritoneal dissection because of extreme fibrosis, ureteric catheters can be used, or the ureter is dissected from the level of the pelvic brim. Catheters enable identification of the ureter by moving the catheter or by feeling the solid consistency of the catheter with a forcep.

Small pinpoint endometriotic lesions can be vaporized using the $CO_2$ laser or unipolar cutting-current held 1–3 mm from the surface of the lesion with resultant drainage of hemosiderin-filled fluid in cases where deposits have progressed to just beneath the peritoneum. The base of the lesion is then vaporized until normal tissue is seen.

The peritoneum tends to be more adherent on the posterior wall of the broad ligament close to the uterus. In this situation knowledge of the course of the ureter and uterine arteries is mandatory. If the lesions are superficial, the peritoneum may be stripped without harming the uterine vessels or ureter.

If the lesion is infiltrative then ureteric dissection from the posterior to anterior lateral pelvic wall determines the position of the uterine artery and vein, crossing the ureter which are then ligated by bipolar electrocoagulation, or clip ligation.

This is an important technique as dissection on the posterior leaf of broad ligament or anterior on the lateral pelvic wall may result in uterine artery bleeding which is difficult to control without risk of damaging the ureter.

When an endometriotic nodule has infiltrated deeply, the anatomy is distorted and the uterine artery or anterior branch of the internal iliac artery is safely ligated close to the pelvic wall. The uterine artery can be found by following the obliterated hypogastric artery posteriorally into the triangle formed by the round ligament, the lateral pelvic wall and the infundibulo-pelvic ligaments. The obliterated hypogastric artery joins the uterine artery deep in the triangle. Using this technique the whole of the peritoneum and lateral pelvic wall can be removed when necessary.

The ovary and/or sigmoid colon may have to be mobilized from the left lateral pelvic wall prior to excision of the peritoneum. If the sigmoid colon is adherent to the pelvic wall and adnexae it is best first mobilized posteriorly close to the junction with the descending colon as correct tissue planes are easier to determine moving from normal to pathologic tissue. Traction of the sigmoid colon (ovary or bowel) should be made with bowel holding forceps, which are flat and a traumatic late bowel perforations have resulted from using tooth forceps holding the bowel wall (not the fat tags on the bowel).

The area of peritoneum excised is also important. Occult (microscopic) endometriosis has been in normal appearing peritoneum in 13–50% of patients [9,11]. Removal of 1–2 cm margin of peritoneum around the lesion may eliminate microscopic foci.

### BLADDER LESIONS

It is difficult to determine the depth of a peritoneal endometriotic lesion on the bladder by inspection. Elevation of the bladder peritoneum is essential. A small incision is made in the normal peritoneum close to the lesion and blunt and sharp dissection used to determine the area and depth of infiltration of the disease process. Aquadissection may facilitate peritoneal separation from the bladder. A metal catheter is helpful in determining the edge of the bladder muscle when excising peritoneal lesions adherent to the bladder. If the lesion extends into muscle of the bladder, cystoscopy is performed to determine the extent and site of bladder involvement. This determines the extent and depth of dissection required and its proximity to the ureteric orifices. The vascularity of the bladder muscle may make it difficult to determine the periphery of the disease process. The edge of the specimen removed requires careful examination to ensure the edge is 5 mm clear of the endometriotic tissue. If the bladder is entered, the hole is closed in 2 layers, with size 1 PDS or monocryla and a catheter is placed *in situ*

for 7 days. A cystogram can be performed after 6 days to confirm healing of the bladder prior to the catheter being removed.

If the bladder lesion is close to a ureteric orifice ureteric catheters are inserted so that the proximity of the endometriosis to the ureteric orifices can be determined. If the disease is close to a ureteric orifice, a urological consultation is sought as reimplantation of the ureter may be necessary.

### ENDOMETRIOMA

There are 4 treatments described:

(1) Drug therapy using GnRH analogues or Danazol®.
(2) Aspiration of the endometrioma with additional anti-endometriosis drug therapy, usually a GnRH analogue or Danazol®.
(3) A three step procedure, laparoscopy to diagnose and aspirate the endometrioma, followed by drug therapy for 3–6 months, followed by re-laparoscopy and removal of the endometrioma.
(4) A one step surgical procedure, excision of the endometrioma.

Removal of the endometrioma is preferable to medical therapy or simple drainage. The short term drug therapy cure rate has ranged from 12% to 45% and the surgical cure rate after 1–3 years from 70% to 90% [15,16]. The best result followed careful excision of endometriomas, 90% of patients being pain free at 1 year and 50% conceived [17].

Aspiration of the contents does not remove the active endometriotic tissue in the wall of the ovary.

Drugs may prevent further growth or reduce the size of the endometrioma. Shrinkage does not indicate shrinkage of the endometriotic tissue, as temporary inactivity in the absence of estrogen stimulation will allow reabsorption of the chocolate fluid in the endometrioma cavity over a period of time. The action of drugs in shrinking the endometriotic lining is limited by 2 things, the difficult access as the lesions are usually surrounded by extensive scar tissue, and the nature of the brown or black lesions, which usually have a low estrogen receptor count, in contrast to red or clear blister lesions seen elsewhere in the pelvis [18].

The 3-step procedure has not proven to be more effective than a single step surgical procedure, the latter having a 90% cure rate after one year follow up [17]. The 3-step procedure assumes that both drainage and drug treatment have lasting benefits, which is very unlikely. In a study of drainage and drug therapy alone, the majority of lesions, 87%, had recurred within one year [15,16]. The only other reason to use the 3-step system is that it may make surgery easier. There is a small advantage in starting the operation with a small ovary, but this advantage no longer exists once the large endometriomas have been drained, which takes only several minutes and reduces the ovary to near normal size. The first 2 steps of the 3-step procedure, drainage by laparoscopy and drug therapy involves unnecessary costs, one unnecessary operation and unnecessary side effects from drug therapy.

The one step procedure has been used successfully in endometriomas up to 16 cm diameter.

### TECHNIQUE OF REMOVAL

The operation is achieved using three accessory incisions: two being used to hold the ovary or the edges of the endometrioma and one for the instrument used to excise the endometrioma. Ports on both sides of the abdomen allow sufficiently wide angles to retract the ovary medially away from the lateral pelvic wall or bowel and to open the endometrioma.

The endometrioma is incised over the point of maximum curvature using electrocautery, laser or scissors. Electrocautery has the advantage of containing bleeding over the line of the incision, while scissors facilitate an excellent view of the ovarian wall and endometrioma capsule adherent to the inner ovarian wall. In a minority of endometriomas an initial incision for drainage is not required, as the endometrioma has formed between the pelvic wall and ovary or between the irregular surfaces of the ovary; the endometrioma drains naturally after mobilization of the ovary.

Drainage is achieved by suction irrigation to reduce spillage of the chocolate-like material found inside the endometrioma. A suction irrigator is placed inside the abdomen at the time of ovarian mobilization, to reduce spillage. Dilution of thick material may be required before the suction is effective.

The cyst cavity is rinsed clean with lactated Ringer's solution and the endometrioma then excised using 5 mm grasping forceps, and a spoon electrode or scissors [2]. To create an initial plane between the ovarian wall and the wall of the endometrioma grasping forceps are placed in 2 parts of the ovarian wall thus opening the cavity of the endometrioma, and a third grasping forceps is used to grasp the capsule of the endometrioma. If this is difficult cutting current (70–100 W), through a scissor or spoon electrode tip is applied at the junction of the cyst wall and ovary to develop a plane of dissection. This step avoids excessive bleeding.

The laparoscope is brought close to the area of dissection, magnifying the wall of the endometrioma clearly. Two grasping forceps to stabilize the ovarian cortex while traction is exerted on the capsule of the endometrioma by a third grasping forceps to peel it from inside the ovary. Sometimes the wall of the endometrioma will not peel away from the ovary, so that scissor, laser or electrocautery excision is required. Thermal injury to the ovary is reduced by using the scissors, with back-up diathermy used only to control bleeding. If the surgical plane for excision is not clear because of excessive scar or bleeding, the wall of the endometrioma may be vaporized by electrocautery or laser. The obvious disadvantage is that thermal injury may occur to adjacent ovarian tissue, reducing the follicle population in the ovary.

Significant bleeding requires the use of bipolar forceps, which reduces the spread of current into the adjacent ovary. Suture of the ovary may also be used to control bleeding, using a 3/0 absorbable suture with a needle large enough to compact the ovarian tissue. If there is a large raw area of ovary, suture may reduce the risk of extensive bowel adherence to the ovary, although controlled trials of routine suture closure of the ovary have shown no reduction of postoperative adhesions compared to leaving an open ovarian wound [19].

Oophorectomy is preferable if no normal ovarian tissue is found after removal of a large endometrioma. Oophorectomy may also be considered if the endometrioma has been recurrent or the ovary is causing pain as a result of being adhered to the lateral pelvic wall by very rigid scar tissue and the ovary fragments when dissected.

### OVARIAN REMOVAL

A preliminary ultrasound is helpful to detail the position of the ovary, its size, associated pathology, and fixity or otherwise to neighbouring structure.

MRI, especially with special coils, is also useful in analyzing details of pelvic organs, including the ovary, to determine the site, size, fixity and associated pathology. In several countries it is more expensive than vaginal ultrasound and is restricted in its use to the diagnosis of the pelvic mass which cannot be assessed by other techniques.

Incisions are made through the umbilicus, 10 mm, and left and right iliac fossa lateral to the rectus muscle, 5 mm. The lateral incisions may be placed higher if the ovary is enlarged above the pelvic brim. Another 5 mm midline incision in the suprapubic area is an advantage in all but the most simple operations. Appropriate trocars are placed through the incisions.

A 10 mm laparoscope is inserted through the umbilicus, and 5 mm grasping forceps, spoon shaped monopolar electrosurgical forceps, bipolar electrosurgical forceps, scissors and a suction irrigator may be placed through the 5 mm incisions.

An intrauterine manipulator is placed inside the uterine cavity.

If the ovary is adherent to the deep pelvis, a rectal probe is placed inside the rectum.

Inspection of the lower abdomen assesses the size and pathology of the ovary, the position of the ovary in relation to the uterus, lateral pelvic wall, and bowel and its fixity or mobility.

If the ovary is mobile it is rotated upwards out of the pelvis by grasping with a tooth or Allis forceps, and the uterus is moved to the side opposite from the ovary being removed, so that the whole of the lateral pelvic wall is visualized, in particular the relationship of the ovary to the ureter [20].

Movement of the ureter confirms its position. If it appears not to move gentle massage or flushing fluid on the ureter may initiate movement. Once the position of the ureter has been established, a further check on its position is made before each step of the operation to avoid possible damage. If the ureter cannot be identified it is visualized at the pelvic brim and its subsequent course determined by freeing the ureter from the peritoneum until it has passed beyond the ovary. If the ureter still cannot be seen, usually due to scarring of the peritoneum from previous endometriosis, the danger of damage to the ureter is increased as its course may be abnormal and the ureter may be stuck on the lower surface of the ovary. The course of the ureter is determined by inserting a ureteric catheter and visualizing the catheter as it is moved up and down. If it still cannot be seen it can be felt by running a blunt instrument across the lateral pelvic wall to feel the catheter.

If the ureter is seen close to or passing over the lower part of the ovary it requires dissection and mobilization. This can be performed by elevating the peritoneum close to but several millimeters above or below the ureter and making a small 5–10 mm incision in the peritoneum (see Lateral Pelvic Wall Dissection).

If the ovary is fixed to the pelvic wall, it may be mobilized by stretching it away from the point of fixity, which allows visualization of adhesions or scar tissue. These are cut after making sure of the course of the ureter. If the ovary if fixed firmly to the pelvic wall, the edge of the ovary may be difficult to determine. A small incision at the edge of the white scar on the retracted ovary, away from the ureter, ovarian blood vessels and the iliac vessels high on the lateral pelvic wall, usually identifies the correct plane for dissection. Once in this plane the dissection usually proceeds rapidly.

Sometimes the ovary is completely retroperitoneal. In this case incision and mobilization of the peritoneum over the ovary will enable definition of the size and position of the ovary. Mobilization of the ovary proceeds quickly with traction and short incisions close to the ovary to avoid the ureter and blood vessels on the lateral pelvic wall. If the ovary cannot be identified incision of the peritoneum lateral to the ovarian vessels and subsequent mobilization of these vessels will lead to the ovary and ureter.

If the ovary is enlarged reducing its size may be helpful prior to removal. Before doing so it is important to make sure the enlargement is not due to cancer or a dermoid cyst. The former may be excluded by vaginal ultrasound, blood tumour markers, such as CA125, CEA and inhibin, and by inspection of the ovary for neoplastic characteristics. If malignancy cannot be excluded it is better to remove the ovary intact even if this can only be achieved by laparotomy. A dermoid cyst is usually diagnosed by characteristic features on ultrasound. If a large dermoid cyst is accidentally ruptured during removal, repeated peritoneal washings with saline or Hartmann's solution, until the peritoneal fluid is clear, will prevent the rare occurrence of chemical peritonitis or implantation of live tissue, e.g. thyroid tissue.

The most common large cysts contain clear fluid, blood, or chocolate coloured thick fluid. These cysts are aspirated by suction irrigation after making a small hole in the ovarian wall. Flushing the inside of the cyst ensures emptying and prevents further spillage during ovarian removal which may slow dissection. Proper deflation of large ovaries enables removal of ovaries as large as a 24 week pregnancy by laparoscopy.

Further mobilization of the ovary may assist removal. This can be done by cutting the ligament attaching the ovary to the uterus using scissors, bipolar, diathermy, or spoon electrode, or laser.

The major ovarian blood supply in the infundibulo-pelvic ligament may be dealt with in 5 ways.

(1) The cheapest method is by suture ligation. The relation of the blood vessels to the ureter is checked. A small hole is made with scissors in the peritoneum below the blood vessels with the ovary stretched away from the pelvic wall to best display the blood vessels. A curved forceps is placed through the hole with a size 1 absorbable stitch, passing lateral to medial below the blood vessels. The end of the suture is grasped by the same forceps placed above the blood vessels, aided by the assistant holding the end of the stitch while the angled forceps takes up the new position above the blood vessels. Two ends of the suture are now outside the abdomen and a knot tied by sliding single knots repeatedly down to the blood vessels, the last two in the opposite loop to lock the knot. Two ligatures are made, each ligature is separated by about 2 cm. The blood vessels are cut between the ligatures.
(2) Bipolar diathermy may be used to close the blood vessels. With large veins or arteries it may be difficult to achieve closure of the vessels with short ended bipolar forceps. Blood flow has to be occluded by the forceps to obtain effective coagulation, so the forceps have to close effectively Kleppinger's corrugated forceps are most effective.
(3) The Harmonic Scalpel® can also be used to close ovarian blood vessels. It has the advantage of the absence of risks of electrosurgery and the absence of smoke. Although the reusable equipment is inexpensive, the basic unit costs AUS $50,000 and about US $32,000.
(4) Vascular clips may be used to close the vessels. They are more expensive and may not be long enough to close large dilated veins.
(5) Staples are the quickest method of closing blood vessels but they are the most expensive. They are easy to use but require either a second 10 mm incision in the lateral part of the abdomen or the use of a 5 mm laparoscope laterally to view the ovary and the placement of the 10–12 mm staple gun through the umbilical incision. The latter is preferable and only requires short experience of looking at the ovary from a different angle.

After the blood vessels are cut the mesovarium attaching the ovary to the lateral pelvic wall requires separation from the ovary. Large veins are present so bipolar forceps or a spoon electrosurgical forceps may be required before the whole ovary is free from its attachments.

The ovary may be removed through the abdomen or vagina.

*The Abdomen*

The ovary may be removed through the 10 mm umbilical port using a 5 mm laparoscope from one of the lateral sites to view the removal. This is important as if a small piece of ovary drops back into the abdomen it may implant and cause subsequent pain. This technique may be aided by:

(a) reducing the size of the ovary using scissors or diathermy to make 2 or 3 separate pieces;
(b) the passage of the ovary through the umbilicus may be aided by placing it in a plastic bag to reduce friction between the ovary and exit during withdrawal, and to allow a strong grasping forceps to be passed into the neck of the bag as it appears in the umbilicus to compress the ovary and increase the force of withdrawal, or;
(c) placing a curved blunt ended scissors into the incision, guided by one finger, and enlarging the umbilical incision by 1–3 cm in a downward direction.

*The Vagina*

If the ovary is too large to remove through the umbilicus or requires to be removed intact (dermoid cyst or possible malignancy) it may be removed through the posterior vaginal vault (posterior colpotomy) [21] or by a transverse suprapubic incision enlarged to the size enabling easy removal.

Removal through the vagina may be done in 2 ways:

(a) by distending the vaginal vault behind the uterus with a sponge forceps, and opening the vagina by laparoscopic diathermy or a laser probe, passing a grasping forceps from the vagina into the pelvis, and extracting the ovary.
(b) by passing a 10 mm trocar and cannula through the vaginal vault, removing the trocar and placing a strong toothed grasping forceps into the low pelvis and withdrawing the ovary. The vagina is more elastic than the abdominal wall and the hole can be easily dilated, and repaired later.

The final step in removal of the ovary is to check haemostasis. Bleeding may be controlled by bipolar forceps near vital structures, or by a spoon monopolar electrode.

Any 10 mm incision requires careful suture of the rectus sheath.

### DEEP PELVIC ENDOMETRIOSIS

In contrast to mild endometriosis, laparoscopy is often unnecessary to diagnose endometriosis in the deep pelvis. It can usually be diagnosed by clinical examination.

These patients need not be subjected to diagnostic laparoscopy, and can be referred to surgeons capable of treating rectal endometriosis [2].

Rectovaginal examination is diagnostic of endometriosis in the pouch of Douglas when nodularity in the rectovaginal septum can be felt and most often pelvic tenderness elicited.

At laparoscopy careful inspection of the pouch of Douglas is necessary to evaluate the extent of adherence of the rectum to the vagina, cervix and uterus [22]. A sponge forceps is inserted into the posterior vaginal fornix and a rectal probe in the rectum. The normal posterior fornix shows a portion of vaginal wall between the cervix and rectum as a distinct and separate bulge. The uterosacral ligaments are normal in calibre, lateral and mobile. Complete obliteration of the pouch of Douglas is diagnosed when the outline of the posterior fornix cannot be visualized initially through the laparoscope; the rectum or fibrotic endometriotic nodules completely obscure the vaginal vault.

### Surgical Technique

Deep pelvic endometriosis involving the pouch of Douglas requires excision of nodular fibrotic tissue from the uterosacral ligaments, posterior cervix, posterior vagina, and the anterior and lateral rectal surface [22,23].

Drug therapy has been less successful in deep pelvic disease, because of the association with extensive scar tissue and the predominance of lesions, brown or black with low levels of estrogen receptors.

The patient is placed in deep Trendelenburg position to allow the small intestines to fall out of the pelvis.

There are 2 main techniques, initial rectal mobilization, or initial lateral dissection of the pararectal space or uterosacral ligaments.

The distinction between vagina and rectal wall may be difficult as the rectum may obscure the vagina and be adherent to it. The vaginal and rectal probes may assist recognition and dissection of the two structures. I prefer to have one finger in the rectum and one finger in the vagina using the left hand and use the right hand to dissect with scissors. Palpation assists visualization both in distinguishing the structures and guiding the dissection and depth of dissection.

The Harmonic Scalpel® may be preferred because of the absence of smoke working deep in the pelvis. If rectovaginal palpation is assisting the dissection, round ended scissors are preferable.

(1) The anterior rectum is dissected from the uterus and vagina until loose areolar tissue in the rectovaginal space is reached. Using the rectal probe or finger in the rectum as a guide to rectal location, the rectal serosa is opened at its junction with the adherent uterus using scissors. Careful sharp and blunt dissection then ensues until the rectum, normal or with contained fibrotic endometriosis is separated from the posterior uterus, cervix, upper vagina, and rectum until surrounding loose areolar tissue is identifiable below the lesion. The fibrotic endometriosis is removed from the posterior vagina, uterosacral ligaments, and rectum only after anterior rectal mobilization is completed.

The ureter is visualized prior to excising the lesion. If it is not seen, dissection at the pelvic brim or lower in the pelvis is required to determine its course. Sometimes the ureter is attached to or surrounded by the endometriotic tissue. An aberrant ureter has been seen medial to the uterosacral ligament. The ureter lies lateral to most cul-de-sac lesions, especially when they are placed on medial traction. With the uterosacral ligament pulled medially, there is less risk of ureteral damage. When the ureter is close to the lesion, its course is traced starting at the pelvic brim, and when necessary, the peritoneum overlying the ureter is opened to confirm ureteral position deep in the pelvis. Uterosacral fibrotic endometriosis may envelop the ureter, necessitating its dissection and excision.

(2) (a) If the uterosacral ligaments are infiltrated with endometriosis, they are removed early in the operation before rectal mobilization. They may make up a large portion of the rectal nodule. Part of the ligament which has a normal calibre is identified on the pelvic side wall, divided and put on traction. The peritoneum is incised on both sides of the ligament, and the thickened portion of the ligament is excised, and including its insertion into the cervix. Normal appearing, soft loose areolar tissue, adipose tissue, uterine vessels, and ureter are found below and lateral to the ligament.

(b) If the lesion is large, fibrotic and planes of dissection are difficult to determine, dissection of the pararectal space may provide a safer and easier approach to defining the anatomy and pathology of rectovaginal endometriosis. The rectovaginal septum often can be defined more easily by a lateral approach to the septum. The fibrotic, often nodular, endometriotic lesions are excised from the uterosacral ligaments, the upper posterior vagina (the location of which is continually confirmed by the sponge in the posterior fornix or finger), and the posterior cervix. The dissection of the fibrotic endometriosis from the vaginal wall proceeds using traction with a biopsy forceps to pull the lesion from one side to the other; electrosurgery or scissors are used.

The extent of vaginal surgery is determined by removing all scarred tissue which is localised by combined vaginal palpation and laparoscopy inspection and instrument palpation. Frequent palpation using rectovaginal examinations helps identify occult lesions. The lesion may penetrate the vaginal wall when dissection removes all visible and palpable fibrotic endometriosis in the vagina.

If the vagina is opened visualization may be maintained by performing gasless laparoscopy using the Maher elevator or by packing the vagina. The gasless technique allows vaginal surgery to be carried out in association with laparoscopic dissection [24].

### RECTAL ENDOMETRIOSIS (R. WOODS [25])

Endometriosis in the pouch of Douglas (POD) can range from simple peritoneal changes to severe infiltrating dense tissue mandating a rectal resection.

Symptoms vary although they are not necessarily proportional to the severity of the pathology. Preoperative assessment should include vaginal and rectal examinations. If able to be performed simultaneously this allows forward placement of any POD lesions and this mobility gives a guide to possible rectal involvement. Diagnosis may require an anaesthetic. A colonoscopy is not routinely performed. Informed consent is crucial and involves discussion of possible bowel resection and potential complications including anastomotic leak and the possibility of temporary stromas.

Cyclical rectal pain, cyclical bleeding and rectal wall involvement seen and/or felt at rectal examination suggests rectal involvement. Preoperative assessment can be difficult and sometimes the full extent is only determined at surgery. If the circumstances are not favourable for complete excision of the endometriosis partial excision should not be attempted as it makes the "definitive" operation more difficult. The patient should be rescheduled.

In difficult POD dissection the dissection is commenced laterally with ureteric dissection and/ or stenting as the uterosacral ligaments are often involved and need excision. Early excision or release of the ligaments allows for maximal anterior–posterior retraction of the uterus/cervix/vagina away from the rectum. Rectal and posterior fornix probes help delineate the rectovaginal plane and demonstrate any fibrosis by placing tissues on traction.

Rectal involvement may be diffuse, localised or in close apposition in increasing order of frequency. The degree of rectal involvement with endometriosis is assessed with a rectal probe and movement of the nodule with a grasper after mobilization of the rectum as described above. If clear of rectal muscle wall it can be easily excised. If the disease is localised over a small area a trial dissection is performed resulting in either total excision or at least debulking of the nodule.

A localised nodule can be managed by laparoscopic excision with suture, transvaginal excision with suture or anterior rectal wall disc excision with a circular stapler. This last option is our preferred approach. This involves debulking the lesion and then placing the circular stapler eccentrically so that the anterior rectal wall with the nodule is contained within the stapler. This is achieved by posterior pressure on the nodule with a suture grasped on either side of the rectum pushing it within the stapler which is applying counter pressure anteriorly. A disc of anterior rectum extending to the midlateral extent is excised.

If diffuse involvement is encountered resection is required. This can be performed laparoscopically with mobilization of the rectum below the endometriosis in the presacral plane and then mobilization of the sigmoid to the midline with identification of the left ureter. The posterior mesorectum (containing the superior rectal vessels) is transected with an endo GIA stapler (vascular) as is the rectum at this level. The sigmoid mesentery is also transected with an endo GIA. The proximal bowel is then delivered through a minilaparotomy and a pursestring suture is inserted into the proximal bowel after transection. The Permian EEA is then fired intracorporeally. Any bowel resection or dissection can also be performed using open laparotomy approach and similar techniques as outlined above.

### ADENOMYOSIS (C. WOOD [26])

Conservative surgery involving endomyometrial ablation, laparoscopic myometrial electrocoagulation or excision has cured symptoms in 32 of 54 patients (81.5%) followed for 3 years [26].

### Technical Difficulty

Laparoscopic surgery may be limited by the need to excise ill-defined tough adenomyotic tissue and to use robust suturing equipment to obtain wound closure after excising significant areas of myometrium. The easier removal of adenomyosis by laparotomy is a less attractive alternative to laparoscopy, particularly as cure cannot be guaranteed. Unless the adenomyosis is well defined, as in an adenomyoma, it is not possible to be certain of cure following excision or electrocoagulation.

The choice of a suitable surgical procedure depends upon the site and extent of disease, the age of the patient, the desire for future pregnancy, the patient's desire for certain cure or not, and the surgical skill of the gynaecologist.

### Endo-myometrial Ablation/Resection

The levonorgestrel IUD may be used to control menorrhagia and dysmenorrhoea. If this fails endometrial ablation may be used.

Endomyometrial resection is most suited to patients with disease limited to the endomyometrial junction as menstrual symptoms may be reduced and the pathology may be removed. It may also be useful when adenomyosis is present in the outer myometrium as laparoscopic myometrial excision alone may not cure menstrual symptoms, either because excision may be incomplete or the menstrual symptoms are not caused by the outer myometrial adenomyosis.

### *Technique*

The technique of endometrial ablation has been well described. If MRI or ultrasound shows the extent and site of endomyometrial distortion the procedure can be modified to include 2–3 mm of myometrium in the affected areas. The whole of the endometrium should be removed as menorrhagia may be due to factors other than the adenomyosis. Deeper myometrial removal or ablation carries the risk of causing increased bleeding as significant arteries are situated about 5 mm deep to the myometrial surface.

### Laparoscopic Myometrial Electrocoagulation

Electrocoagulation has the capability of shrinking adenomyosis by causing necrosis. The technique has been applied to localized or extensive disease. The adenomyosis can be detected by MRI, vaginal ultrasound, inspection of the uterus at laparoscopy, myometrial needling, or manual palpation during gasless laparoscopy to detect differences in consistency between normal and abnormal tissue. Electrocoagulation may reduce the strength of the myometrium by replacing abnormal myometrium with scar tissue. The width of the scar is more extensive than after surgical excision when close apposition of normal myometrium is achieved.

Electrocoagulation is best suited to women over 40 years of age, who do not wish to conceive, and who wish to avoid more extensive surgery such as excision or hysterectomy. Even if recurrence occurs the procedure may be repeated until the onset of the menopause when symptoms cease.

### *Technique*

Uterine manipulation with a Valtchev manipulator improves access to the diseased areas by facilitating antero-posterior and lateral movement of the uterus.

Vasoconstricting agents such as adrenaline and vasopressin are not used routinely as excessive bleeding has not been experienced and the blanching of the myometrium after vasoconstriction makes it difficult to determine the devascularizing effect of electrocoagulation or uterine vessel closure.

Closure of the ascending uterine artery may be performed if future pregnancy is not wanted, and the site of the adenomyosis is in the upper uterine body. Bipolar forceps, clips or suture ligation may be used to close the uterine vessels. Laparoscopic uterine artery ligation may also be achieved lateral in the pelvis after dissection of the ureter.

Electrocoagulation of the adenomyosis may be carried out with unipolar or bipolar needles, using 50 W coagulation current. Bipolar needles have a theoretical advantage of concentrating current between the two needles, but their effectiveness is diminished by the tendency of the two needles to move close together as they penetrate the myometrium. Additionally, the area of coagulation may spread outwards from each needle, simulating the effect of monopolar electrocoagulation.

The extent of coagulation can be controlled by reducing the current strength and changing the time the needle(s) are held in position. In order to reduce the possibility of severe surface necrosis and carbonization, either of which may encourage future adhesion formation, the insulated part of the needle is buried a few millimetres below the uterine surface before electrocoagulation is commenced. The insulation on the bipolar needle can be extended so that the active part of the electrode is shortened in order to avoid surface coagulation and necrosis. Needle punctures are made at 1–2 cm intervals, depending on the spread of the coagulative effect. The depth of needle puncture may vary, depending on the thickness of the adenomyotic myometrium. This varies from 3–25 mm.

If hysteroscopic endomyometrial ablation has also been carried out, the depth of laparoscopic needle electrocoagulation may be reduced. Laser has been used to shrink fibroids but its use has not been reported in the laparoscopic treatment of adenomyosis.

Electrocoagulation is best suited to women over 40 years of age, who do not wish to conceive, and who wish to avoid more extensive surgery such as excision or hysterectomy. Even if recurrence occurs the procedure may be repeated until the onset of the menopause when symptoms cease.

### Myometrial Excision

Adenomyosis may be excised if it does not involve the major portion of the uterus. The technique is most suitable for adenomyomas where the margins of the pathology are more easily defined. It may be useful in women wishing to become pregnant, providing sufficient myometrium remains to allow uterine expansion and term pregnancy and the scar formed after excision is not wide or shallow. MRI or colour doppler ultrasound after surgery should be used to check both for cure, the width and depth of scar, and the possible association of residual adenomyosis close to the scar, before attempts at conception are advised.

### *Technique*

Preoperative GnRH analogues or Danazol® may reduce uterine vascularity, correct anaemia if the patient has severe menorrhagia, and reduce operative bleeding which facilitates surgery by laparoscopy rather than laparotomy. Vasoconstrictor drugs may also reduce bleeding at the time of surgery.

Prior to myometrial excision, as with electrosurgical coagulation, the uterine blood supply may be reduced by suture or clip ligation or bipolar diathermy of the uterine vessels in women not concerned with fertility. Apart from reducing bleeding during surgery the reduction in blood flow may reduce future growth or development of adenomyosis.

Two associated surgical procedures may be offered, sterilization to prevent conception and hysteroscopic endomyometrial ablation if menorrhagia is present, and fertility is not required.

Laparoscopy, and gasless laparoscopy, with or without minilaparotomy, facilitate myometrial excision avoiding the need to perform laparotomy. Gasless laparoscopy is done with an abdominal elevator, the Maher elevator forming an S-shaped loop, being effective and cheap [24]. A finger or laparotomy instruments can gain entry to the abdomen through a 2–4 cm incision which may be sufficient to remove and repair areas of myometrium up to 6 × 8 cm.

A Valtchev uterine manipulator is used to position the adenomyotic areas as close as possible to a laparoscopic or minilaparotomy incision. Sometimes a myoma screw may stabilize the diseased area and aid excision. A diathermy spoon using 100 W monopolar current, or scalpel, are suitable for excision. The spoon has the advantage of cutting effectively with the sharp end close to the tissue, and coagulating vessels when the convex curve of the spoon compresses the vessel. When the tissue is very firm the scalpel may be preferable, providing more effective and rapid excision. The scalpel can be used safely through a 2 cm accessory laparoscopy incision or a minilaparotomy incision. The margin of the adenomyosis may be determined by change in appearance, vascularity or consistency; finger palpation may be an advantage.

A myometrial morcellator may also be used to remove adenomyotic tissue coring pieces up to 15–20 mm in diameter. The difficulty in definition of the margin of adenomyotic tissue make morcellation less precise than scissor or knife dissection. The morcellator hides the tissue as it is cored out. The risk of trauma to other organs is prevented by the myometrium being drawn outwards or the instrument not being inserted beyond the surface of the uterus. The morcellator may be hand or electrical driven. It costs AUS $7000–12,000. Lateral insertion in the abdominal wall is essential for safety. A 10 mm laparoscope gives a better view of the procedure. Laparoscopic or gasless laparoscopy, using a large scalpel blade and/or large heavy scissorsto morcellate adenomyosis as it is withdrawn from a small 2–4 cm incision in the umbilicus, the suprapubic area, or vagina, may be a better technique [26]. This technique is cheaper than a morcellator, is just as quick, and may be safer as the surgery is done under vision in the abdominal wound.

Closure of uterine incisions longer than 5–6 cm may require laparotomy instruments as excision of a significant volume of myometrium increases the tension at the myometrial edges which may have to be stretched to close the defect. If the uterine wound is brought into a minilaparotomy incision, the defect can be closed more easily and quickly. Absorbable sutures (No. 1) are used in one or more layers. If there is a large defect a single layer through and through suture may best approximate the wound, acting as a tension suture, and because of the increased thickness of the whole myometrium, it is less likely to tear as tension is increased to attain closure. In 2 patients, 30% and 50% of the posterior myometrium has been removed and allowed successful completion of pregnancy at 34 and 36 weeks [26].

Anti-adhesives such as Interceed® and Goretex® membrane may be used [27–30].

Interceed may be used if perfect haemostasis is obtained. Application of Surgicel® prior to Interceed may improve haemostasis and allow the use of Interceed. If bleeding persists Goretex can be stapled over the wound. This need not be removed unless pregnancy is planned. Uterine enlargement may displace the membrane from the uterus which may attach to other organs.

### CONCLUSIONS

The techniques described have been proven to be safe and effective over 5 years, involving 650 (CW) patients with endometriosis and adenomyosis. No serious complications such as ureteric trauma or undiagnosed opening of the bowel or bladder have occurred. Entry into the bowel or bladder has only occurred when excising endometriosis in these organs.

The one year cure of symptoms for the various types of endometriosis are between 75% and 90%. In a smaller sample of 56 women with recurrent endometriosis, the 2–3 year cure rate has been 78%. The limitations of thermal ablation are evident: the destruction of lesions without biopsy involves a false positive diagnosis in 20–50% of lesions diagnosed visually and the limitation of removing infiltrating lesions over the bladder, bowel and ureter.

The current surgical techniques are based upon the proper identification of all macroscopic disease, consideration of the presence of microscopic disease, and excision in a manner which copies the principles involved in removal of malignant disease.

Further consideration of laparoscopic surgical anatomy, and functional histopathology of the endometriosis and the behaviour of various types of endometriosis can be expected to modify current surgical techniques.

### *Acknowledgements*

H. Reich and D. Redwine have developed some of the techniques described and assisted myself and Peter Maher copy their techniques. The script concerning endometriosis was written by C. Wood and resulted from a large experience of endometriosis surgery performed by P. Maher and myself working together and separately which facilitated both collaboration, constructive criticism and sharing of experience and ideas. A wide variety of techniques are described, which we have used, and enable surgeons to choose techniques to suit their own skills, available equipment and cost restraints. The rectal surgery was described and performed by R. Woods, bowel surgeon, who has a large experience of rectal endometriosis. The adenomyosis surgery was described and performed by C. Wood.

### *References*

[1] Wood, C. Radical laparoscopic surgery for treatment of endometriosis. In: Minaguchi, H. and Sugimoto, O. (Eds.). Endometriosis today, advances in research and practice. *Proceedings of the Vth World Congress of Endometriosis*, Yokohama, Japan, 1996: pp. 289–296.

[2] Reich, H. Laparoscopic surgery for advanced endometriosis. Internet article, http:/www.womensurgerygroup.com/ Laparoscopic Surgery for Adv. Htm.

[3] Metzgar, D.A., Olive, D.L. and Haney, A.F. Limited hormonal responsiveness of ectopc endometrium. Histologic correlation with intrauterine endometrium. *Hum. Path.* 1988; **19**: 1417–1424.

[4] Wood, C. Endoscopy in the management of endometriosis. In: Wood, C. (Ed.) *Gynaecological Operative Laparoscopy: Current Statues and Future Development, Clinical Obstetrics and Gynaecology* 1994, Baillere Tindall, London, pp. 735–757.

[5] Reich, H., Clarke, C. and Sekel, L. A simple method for ligating with straight and curved needles in operative laparoscopy. *Obstet. Gynecol.* 1992; **79**: 143–147.

[6] Jansen, R.P.S. and Russell, P. Non pigmental endometriosis: clinical, laparoscopic and pathological distribution. *Am. J. Obstet. Gynecol.* 1986; **155**: 1154–1159.

[7] Cook, A.S. and Rock, J.A. Diagnosis of endometriosis: laparoscopic appearances. In: Sutton, C. and Diamond, M. (Eds.). *Endoscopic Surgery for Gynaecologists*. London: Saunders, 1993: p. 202.

[8] Martin, D.C., Hubert, C.P., Vander Zwaag, R. *et al.* Laparoscopic appearances of peritoneal endometriosis. *Fert. Steril.* 1989; **51**: 63067.

[9] Martin, D.C., Armic, R. and El Sehg, F.A. Histologic diagnosis of endometriosis. *J. Gynecol. Surg.* 1990; **6**: 275–279.

[10] Wood, C. Histology of multiple lesions present in 30 consecutive patients having laparoscopic peritoneal excision, 1999 (Unpublished data).

[11] Nissole, M., Caspanas-Roux, F. and Donnez, J. Histologic study of occult endometriosis after hormonal therapy. *Fert. Steril.* 1988; **49**: 423–426.

[12] Redwine, D.B. Conservative laparoscopic excision of endometriosis by sharp dissection: life table analysis of reoperation and persistent or recurrent disease. *Fert. Steril.* 1991; **56**(4): 628–634.

[13] Wheeler, J.M. and Malseak, L.R. Recurrent endometriosis: incidence, management and prognosis. *Am. J. Obstet. Gynecol.* 1983; **146**: 247–253.

[14] Winkel, C.A. and Bray, M. Use of leuprolide acetate in combination with surgical treatment for women with endometriosis. In: Minaguchi H. and Sugimoto O. (eds.). *Endometriosis Today Advances in Research and Practice* 1996; pp. 365–369.

[15] Canis, M. Surgery of the ovary. Presented at the 3rd Meeting of the International Society for Gynaecologic Endoscopy, Washington June 24–26, 1993.

[16] Dequesne, J. $CO_2$ laser laparoscopy for peritoneal and ovarian endometriosis. Abstracts of 3rd World Congress on Endometriosis (018), Brussels, Belgium 1–3 June 1992.

[17] Wood, C., Maher, P. and Hill, D. Diagnosis and surgical management of endometriomas, *Aust. NZ J. Obstet. Gynaecol.* 1992; **32**(2): 161–163.

[18] Nissole, M., Donnez, J. and Casanas-Roux, F. Expression of steroid receptors, vimentin and cytokeratin expression in endometriotic tissue. In: Minaguchi, H. and Sugimoto, O. (eds.) *Proceedings of the Vth World Congress on Endometriosis*. Yokohama, Japan: 1996; p. 165.

[19] Nezhat, C. and Nezhat, F. Postoperative adhesion formation after ovarian cystectomy with and without ovarian reconstruction. 47th Annual Meeting of the American Fertility Society, Orlando, Fl, October 21–24, 1991.

[20] Reich, H. and McGlynn. Laparoscopic oophorectomy and salpingo-oophorectomy in the treatment of benign tubo-ovarian disease. *J. Reprod. Med.* 1986; **31**: 609–611.

[21] Wood, C., Hill, D. and Maher, P. Laparoscopic culdotomy. *Aust. NZ J. Obstet. Gynaecol.* 1993; **33**(1): 67–70.

[22] Reich, H., McGlynn, F. and Salvat, J. Laparoscopic treatment of cul-de-sac obliteration secondary to retrocervical deep fibrotic endometriosis. *J. Reprod. Med.* 1991; **36**: 516–522.

[23] Wood, C., Maher, P. and Hill, D. Laparoscopic Removal of Endometriosis in the Pouch of Douglas. *Aust. NZ J. Obstet. Gynaecol.* 1993; **33**(3): 295–299.

[24] Maher, P., Wood, C. and Hill, D. Excision of endometriosis in the pouch of Douglas by combined laparovaginal surgery using the Maher abdominal elevator. *J. AAGL* 1995; **2**(2): 199–202.

[25] Woods, R. Surgical management of rectal endometriosis: prepared for this article.

[26] Wood, C. Surgical and medical treatment of adenomyosis. *Hum. Reprod. Update* 1998; **4**(4): 323–336.

[27] Diamond, M.P., Daniell, J.F., Feste, J. *et al.*, Adhesion reformation and de novo adhesion formation after reproductive pelvic surgery. *Fertil. Steril.* 1987; **47**(5): 864–66.

[28] Operative Laparoscopy Study Group. Postoperative adhesion development after operative laparoscopy: evaluation at early second-look procedures. *Fertil. Steril.* 1991; **55**(4): 700–4.

[29] Jansen, R.P.S. Prevention of pelvic peritoneal adhesions. *Current Opinion in Obstet. Gynecol.* 1991; **3**: 369–74.

[30] Bulletti, C., Polli, V., Negrini, V. *et al.*, Adhesion formation after laparoscopy myomectomy. *JAAGL* 1996; **3**(4): 533–6.

